# Dental autotransplantation as a alternative treatment for the loss of permanent anterior teeth in children

**DOI:** 10.1590/2177-6709.27.4.e22spe4

**Published:** 2022-09-23

**Authors:** Marcos Flávio Spínola AMBRÓSIO, Renata Pittella CANÇADO, Bruna Carolina Gonçalves de OLIVEIRA, Marco Antônio MASIOLI, Deise Lima CUNHA

**Affiliations:** 1 Universidade Federal do Espírito Santo, Departamento de Clínica Odontológica (Vitória/ES, Brazil).; 2 Universidade Federal do Espírito Santo, Curso de Graduação em Odontologia (Vitória/ES, Brazil).; 3 Universidade Federal do Espírito Santo, Departamento de Prótese Dentária (Vitória/ES, Brazil).; 4 Associação Brasileira de Odontologia - Seção Espírito Santo, Curso de Especialização em Ortodontia (Vitória/ES, Brazil).

**Keywords:** 3D computer-aided imaging, Autotransplantation, Dental trauma

## Abstract

**Introduction:**

Autotransplantation is defined as the surgical movement or transposition of a tooth from its original site to a recipient alveolus, in the same patient. It has high success rates when performed within predefined parameters.

**Objective:**

This study aims to describe the advantages of a dental autotransplantation protocol based on a multidisciplinary approach and using cone beam computed tomography, computer-aided planning, and rapid prototyping of the donor tooth, enabling the preparation of a surgical guide and postoperative protective plate. This article discusses the indications and contraindications for autotransplantation, as well as the selection criteria for the tooth to be transplanted and the transoperative care essential for its success. The parameters for post-operative control are described, in addition to the variables of success and failure to be considered.

**Conclusions:**

When analyzing the treatment options available for children with anterior tooth loss and the psychosocial impact on these patients, autotransplantation is considered not only an alternative treatment, but the only viable option for their functional, aesthetic, and social reestablishment.

## RESUMO

**Introdução:**

O autotransplante é definido como a transposição cirúrgica de um dente do seu local original para um alvéolo receptor no mesmo indivíduo, e apresenta altas taxas de sucesso, quando realizado dentro de parâmetros predefinidos.

**Objetivo:**

O presente estudo tem como objetivo descrever as vantagens de um protocolo de autotransplante dentário baseado em uma abordagem multidisciplinar e utilizando tomografia de feixe cônico, planejamento assistido por computador e prototipagem rápida do dente doador - possibilitando a confecção de um guia cirúrgico e placa protetora pós-operatória. Nesse artigo, serão discutidas as indicações e contraindicações do autotransplante, assim como os critérios de seleção do dente a ser transplantado e os cuidados transoperatórios essenciais para o seu sucesso. Serão descritos os parâmetros para controle pós-transplante, assim como as variáveis de sucesso e insucesso a serem consideradas.

**Conclusão:**

Ao analisar as opções de tratamento disponíveis para crianças com perdas dentárias na região anterior e o impacto psicossocial nesses indivíduos, deve-se considerar que o autotransplante não se constitui somente em uma alternativa de tratamento, mas sim na única opção viável para o restabelecimento funcional, estético e social desses pacientes, reinserindo-os em seu meio.

## INTRODUCTION

Autotransplantation is defined as the surgical movement or transposition of a donor tooth from its original site to a recipient alveolus in the same patient.

This process consists of a biological procedure in which teeth, especially in the germinative phase, have the ability to enhance and induce alveolar bone growth,^1^ thus allowing the restoration of aesthetics and balance of the oral cavity by means of a natural tooth of the patient.^2^

**Figure 1:**
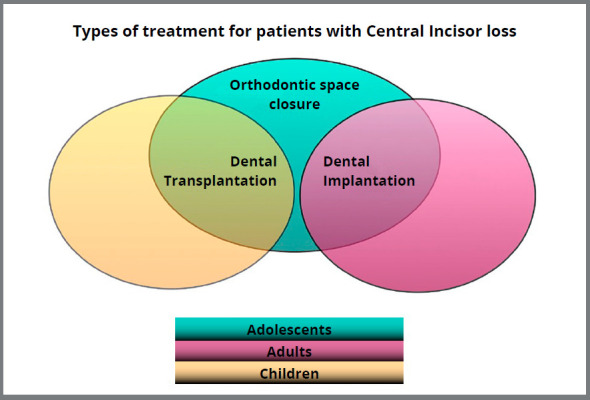
Ideal indications for replacement of lost dental elements.

In an area of tooth loss, the initial possibilities for the replacement of this element would be: osseointegrated implants, fixed or removable prostheses, orthodontic space closure and dental autotransplantation. Each of these options has its indication and recommended timing for execution (Fig 1). 

Autotransplantation is considered a good treatment option in cases of tooth loss due to trauma or anodontia in growing patients.^3^ In addition to dental autotransplantation, the rehabilitation of a pediatric patient with tooth loss can be done orthodontically, by closing the space corresponding to the lost tooth. Another option would be the use of removable prostheses, or prostheses over temporary implants. In cases where space closure is performed, permanent aesthetic limitations will occur. The use of removable prostheses has been proven to lead to poor quality of life in individuals in this age group. Treatment involving dental implants become limited by the continuous dentoalveolar development of patients in active growth phase, since the implants do not follow the eruption of neighboring teeth.^4^ Consequently, autotransplantation becomes an interesting alternative for these patients.

The differences between dental transplants and dental implants are cited in Table 1.

**Table 1:** Differences between dental transplants and dental implants

Dental transplants	Dental implants
Biological substitution	Artificial replacement
Able to create alveolar bone	Requires bone grafting
Normal periodontium	Rapid ankylosis of the osseointegrated implant
Orthodontic movement	Cannot be moved
Erupts like a normal tooth	Does not erupt as a normal tooth
Keeps the tooth papilla intact	Altered tooth papilla

Source: Zachrisson^27^, 2002.

Dental trauma is the second most frequent cause of pediatric dental consultation.^3^ Bauss et al.^5^ reported the incidence of trauma in permanent incisors and found that 10.3% of their sample had a history of trauma on these teeth. The highest prevalence of dental trauma occurred between 11 and 15 years of age, and most of them were in the mixed dentition. In this study, 79.6% of the teeth affected by trauma were maxillary central incisors. The authors reported a statistically significant prevalence of trauma to these teeth in patients with increased overjet, with or without adequate lip coverage. This becomes extremely relevant when we note that the Brazilian population has a prevalence between 36 to 40% of dental Class II individuals^6^ ^,^ ^7^.

In addition to altered physiognomy, patients affected by missing anterior teeth routinely present malocclusions associated with such absences.^8^ ^,^ ^9^ ^,^ ^10^

The success of autotransplantation is quite sensitive in relation to its indication and technique. This sensitivity has caused this procedure to go through moments of popularity and oblivion throughout history. The multidisciplinary treatment with the participation of an endodontist, a surgeon, a professional of aesthetics and an orthodontist increases the rate of satisfaction with the treatment, because it leads to a result both functionally and aesthetically satisfactory.

In this context, patients with missing teeth can undergo dental autotransplantation, but attention should be paid to the correct indication of the procedure. Important factors must be evaluated, such as the availability of a tooth for autotransplantation, the presence of space for rehabilitation, the presence of alveolar bone tissue in the recipient site, the stage of development of the tooth to be autotransplanted, and the general and oral health status of the patient. 

Dental autotransplantation is also a solution for teeth in which endodontic treatment has failed, since the primary goal of endodontic treatment is the prevention and resolution of pulpal and periapical pathologies, to restore healthy peri-radicular tissues.^4^ ^,^ ^11^ Often traumatized teeth enter endodontic processes that indicate resorption, either by replacement or inflammatory. The decision to indicate the extraction of this tooth and perform an autotransplantation can be mistakenly postponed by the professional, and this reluctance can make the execution of this technique unfeasible, by losing the ideal moment of the donor tooth roots formation (Nolla stage).

**Figure 2:**
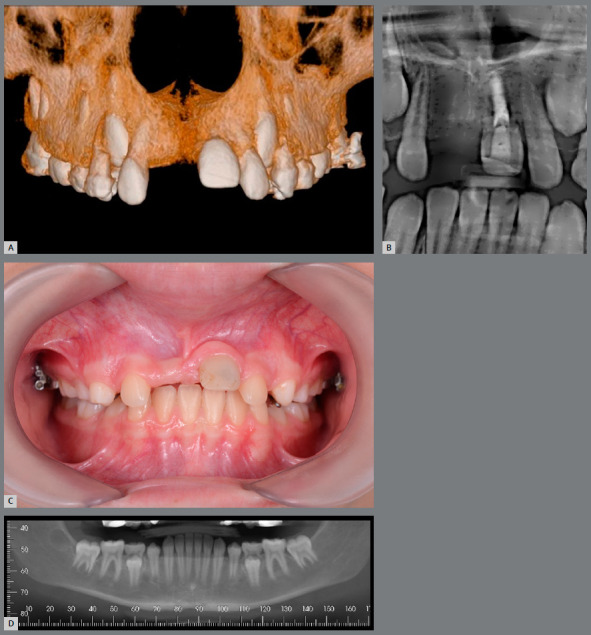
Patient with missing right maxillary central incisor (#11)**(**A), left maxillary central incisor (#21) with root canal treatment **(**B), presenting replacement resorption. Donor tooth in the ideal period for carrying out the transplantation **(C**, D).

Regarding the surgical technique, the chronological and dental age of patients also play a role in its success. The definition of the donor tooth selected for autotransplantation should preferably involve teeth with an open apex, with a root still in development, in Nolla stage 8, which is the ideal indication for autotransplantation. The choice of tooth is made by means of an individual assessment of each case, conducted jointly by the surgeon and orthodontist during the virtual planning, taking into account the tooth shape and dimensions, the anatomy and configuration of the recipient site, and the patient’s occlusal pattern, in addition to planning the orthodontic treatment after transplantation (Fig 2).

During the surgical procedure, the recipient alveolus should be created 10% larger than the size of the donor tooth. The procedure for extracting this tooth should be as atraumatic as possible, without damaging the dental cementum and preserving the periodontal ligament fibers. The time between extraction of the donor tooth and its placement in the recipient site should be as short as possible. This tooth should be placed in infraocclusion, and a flexible retention made with suture thread should be performed (Tables 2A, 2B and 2C).

**Table 2A:** Essential requirements for surgical procedure success.

A	Requirements	Justification	
I	Tooth germ at Nolla’s stage 8	For the re-establishment of dental vitality, as well as root development	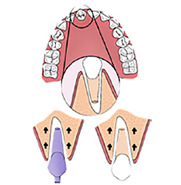
II	Alveolus 10% larger than the transplanted tooth	Avoids pressure on the transplanted tooth, using a prototype as a template	
III	Shortest possible time out of the alveolus	With the use of the printed prototype of the donor tooth, it is possible to prepare the alveolus before extracting the transplanted tooth from its original site	

**Table 2B:** Essential requirements for the success of the surgical procedure.

B	Requirements	Justification
IV	Periodontal ligament cannot be injured	The cells of the periodontal ligament, through its differentiation, will be responsible for healing of the tooth in the alveolus
V	Preserving the cementum	Avoids the risk of internal resorption
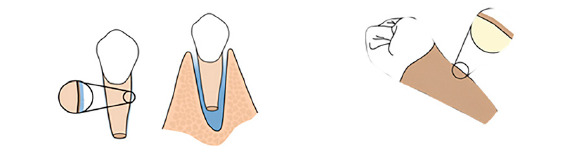		

**Table 2C:** Essential requirements for the success of the surgical procedure.

C	Requirements	Justification
VI	Semi-rigid retention	Allows physiological mobility, thus avoiding islands of ankylosis
VII	The transplanted element needs to be placed in infraocclusion	Under no circumstances should the transplanted element suffer any type of occlusal trauma
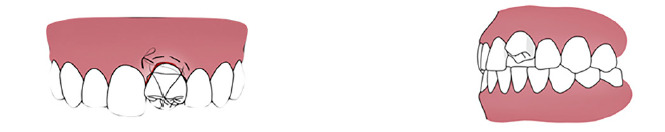		

The observation of each of these stages of diagnosis and surgical procedure is essential for the survival and success of the transplanted tooth. Immediate postoperative care should also be performed, with tooth protection and dietary restriction.

Dental autotransplantation offers multiple advantages, especially for young patients in growth period, since it is done with autologous tissue, preserves the periodontal ligament, volume and bone morphology, allowing continued skeletal growth, functional adaptation, proprioception, resistance to occlusal loading, improves chewing, maintains integrity of the space, and allows for good aesthetic results, due to maintenance of the naturally adhered gingiva, when compared to other techniques. In addition, wear of adjacent teeth for prosthetics is avoided. Movement with orthodontic appliances is also possible, preserving alveolar bone structure and recovering the lost tooth space, translating into better aesthetics, chewing, speech and dental arch integrity.^11^ ^,^ ^12^

Rohof et al.^8^ stated that current literature evidence on autotransplantation of teeth with incomplete root formation shows favorable survival and success rates, with low complication rates, indicating that it is a reliable treatment option. However, complications do exist, and the most common after autotransplantation include: ankylosis, root resorption, pulpal necrosis, and hypermobility.^8^ ^,^ ^13^

According to Ravi Kumar et al.,^14^ the most important criterion for success involving the recipient site is the adequacy of bone support. There should be sufficient alveolar bone support in all dimensions, with adequate adhered keratinized tissue, to allow stabilization of the transplanted tooth. In addition, the recipient site must be free of acute infection and chronic inflammation.

The planning of dental autotransplantation is not just an isolated surgical procedure after a tooth loss, but is within a global orthodontic planning aiming at a stable and aesthetic occlusion in the long term. Moreover, unlike other treatment approaches, autotransplanted teeth still have the capacity for functional adaptation.^15^ ^,^ ^16^

Recently, image capture using cone beam computed tomography (CBCT), intraoral scanning, digital planning of the entire surgery, the printing of three-dimensional models, preparation of surgical guide and tooth prototype, and the acetate protective plate were introduced, separately or in different associations. These methods aim for better adaptation, reduced damage to the periodontal ligament, shorter surgery time and, consequently, reduced time of the transplanted tooth outside the alveolus. 

Thus, the objective of this article is to present a virtual planning protocol for autotransplantation in children victim of trauma. 

### PROTOCOL

All patients should initially undergo a clinical examination including anamnesis, history of trauma, intra and extraoral physical examination. Them, an orthodontic documentation is requested, including periapical radiographs, panoramic and profile cephalometric radiographs, intra and extraoral photographs, full CBCT and intraoral scanning. 

From this documentation, initially a facial and dental diagnosis of the patient is performed, identifying malocclusions and the need or not for treatment. The identification of the individual’s facial and dental pattern will help in determining which tooth will be used for autotransplantation, since the extraction of this tooth will be part of a global orthodontic treatment plan. Not understanding this essential premise for a successful autotransplantation has been a ongoing cause of dissatisfaction with autotransplanted teeth in the past. 

**Figure 3:**
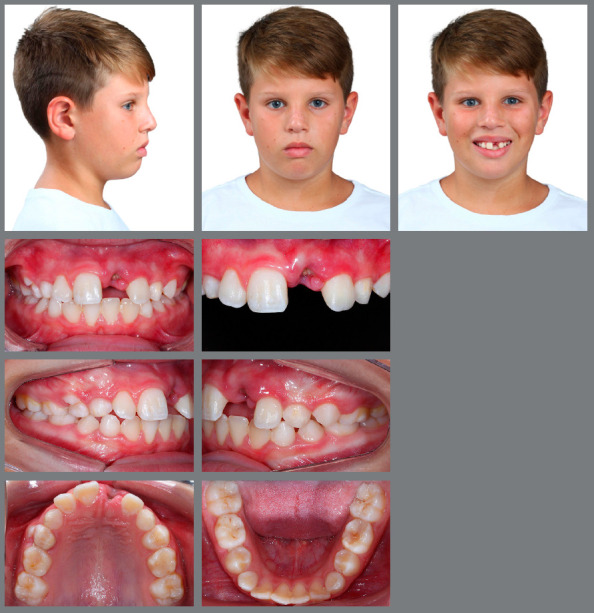
Initial extraoral and intraoral photographs.

**Figure 4:**
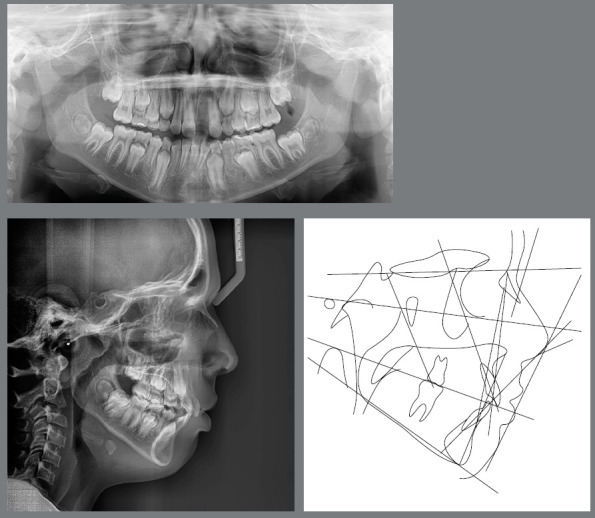
Initial radiographs and cephalometric tracing.

Now it will be reported, for illustrative purposes, the clinical case of a male patient, 11 years and 3 months old, with history of tooth #21 avulsion, due to a fall from a toboggan in a water park. He was referred to a dental clinic, where it was decided to perform dental autotransplantation, since the reimplantation was not possible because the tooth was not found (Figs 3 and 4, Table 3).

**Table 3:** Cephalometric measurements.

Cephalometric measurements		
	Pretreatment	Normal
Maxilla to cranial base		
SNA	81º	82 ? 2º
Mandible to cranial base		
SNB	76º	80 ? 2º
Sn.GoGn	35º	32º
FMA (MP-FH)	27.5º	25º
Maxillomandibular		
ANB	5º	2 ? 2º
Wits	- 0.7 mm	0/-1 mm
Maxillary dentition		
1-NA	4 mm	4 mm
1.NA	26º	22º
Mandibular dentition		
1-NB	5.5 mm	4 mm
1.NB	32º	25º
IMPA	98º	87 ? 6º
Soft tissues		
Lower lip to E-plane	-1 mm	
Upper lip to E-plane	1.4 mm	

### DIAGNOSIS AND ORTHODONTIC TREATMENT PLAN

The patient presented a Class II in Steiner analysis, with well positioned maxillary incisor and protruded mandibular incisors. He presented vertical growth pattern, with a balanced facial pattern and mandibular second premolars with root development at Nolla’s stage 8. By means of the evaluation of the orthodontic documentation and the -2 discrepancy of models by Moyers analysis, the patient had no indication for premolar extraction for orthodontic treatment. Therefore, the premolar chosen for autotransplantation was based on the ideal morphological characteristics of the tooth to be replaced.

### VIRTUAL PLANNING

For virtual planning, full CBCT was performed, scan volumes were exported in DICOM 3 format and imported into image analysis software, where interactive processing tools were used to remove image artifacts. 

**Figure 5:**
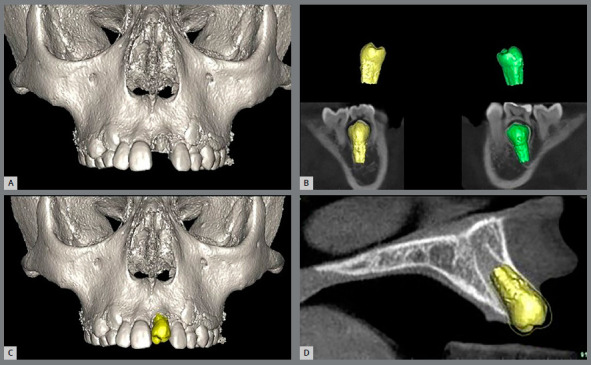
Initial tomography of the maxilla **(**A). Segmentation of possible elements to be transplanted **(**B). Positioning of the chosen dental element in the alveolus of the lost tooth **(C,** D).

**Figure 6:**
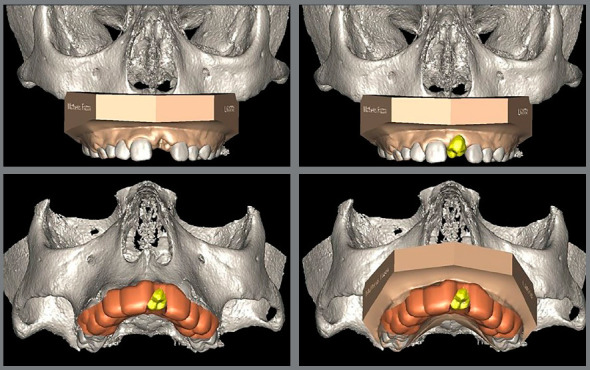
Superimposition of maxillary CBCT, intraoral scan and segmented dental element chosen, for the construction of the surgical guide.

Subsequently, a 3D surface mesh of the donor tooth and the recipient site was created and stored as a standard triangulation language file (segmented stereolithography, STL) (Fig 5). These files were imported into VistaDent 3D Pro 2.1 software. In the segmentation mode, the premolars that could be chosen as donor tooth were evaluated by the orthodontist and surgeon, and together with the STL file of the recipient site and the iTero Element Scanner ^®^intraoral scan file, were transferred to the planning mode, so that accurate guides could be fabricated (Fig 6).

The correct angulation, rotation and precise positioning of the donor tooth in the recipient area was predefined. Its exact 3D position was defined in relation to the anatomical space, adjacent teeth and occlusion, as well as a spatial relationship to ensure an esthetically ideal and functional restoration after the surgical procedure.

**Figure 7:**
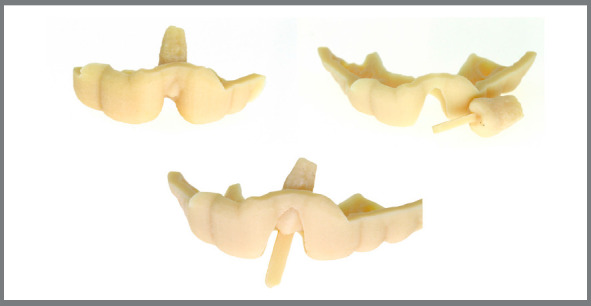
Printed surgical guide and prototype of the tooth to be transplanted.

**Figure 8:**
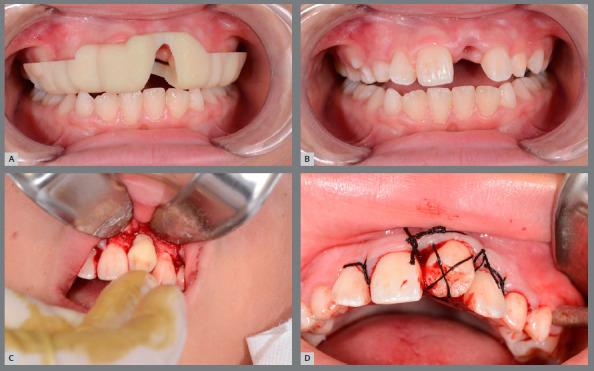
A) Proof of the surgical guide. B) Making the alveolus. C) Proof of the prototype in the prepared alveolus. D) Transplanted tooth into position and stabilized.

Thus, initially a presurgical intraoral scan of the patient’s dental arch was obtained, together with digital planning, aided by a CBCT scan, to analyze the volumetric size of the donor tooth. This analysis was performed for teeth #35 and #45, to select the most appropriate element for the recipient alveolus. As previously reported, both elements were in Nolla’s stage 8, therefore, with a degree of root development suitable for treatment. The adaptation of the teeth prototypes was tested in the target alveolus using the planning software, and the prototype of tooth #45 proved to be more effective, and was therefore selected. Subsequently, the surgical guide was made and tested, which was used during the transoperative phase (Figs 7 and 8).

### SURGICAL PROCEDURE

The surgical procedure was performed with the prior use of antibiotics (amoxicillin), initiated in double dose appropriate to the patient’s weight in the preoperative period, and maintained for 7 postoperative days in its recommended therapeutic dosage. Non-steroidal anti-inflammatory drugs were also prescribed in the preoperative period, starting 1 hour before the procedure and maintained for 3 postoperative days. Analgesics were prescribed only in the postoperative period for 24 hours. 

The surgical procedure began after local anesthesia in the recipient site, where access to the bone structure was performed through flap and mucoperiosteal detachment. At this time, the surgical guide was used with the intention of adapting the recipient alveolus to the angulation, rotation and positioning predefined in the virtual planning. After these data had been transferred, the prototype of the donor tooth supported by an occlusal arch was taken to the alveolus so that we could obtain a space approximately 10% larger than the donor tooth, without it being placed under pressure in the alveolus. With the recipient site prepared, the donor tooth was extracted. The surgery for extraction of the donor tooth was performed atraumatically, with the aim of preserving the tooth structure, by performing a linear flap over the ridge, a small osteotomy and extraction with elevators. 

The donor tooth was then immediately transferred to the previously prepared recipient site, and placed in infraocclusion. Sutures over the occlusal plane of the implanted tooth were performed as a form of postoperative stabilization and non-rigid fixation. These sutures were removed 15 days after the procedure.

**Figure 9:**
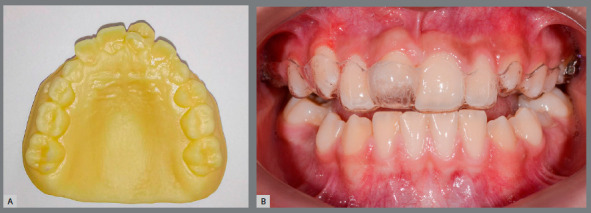
Impression of the post-transplantation model **(**A), for making the protection plate **(**B).

The day after the surgical procedure, a new intraoral scan was performed and, from it, a model of the maxilla was printed containing the donor tooth in its recipient site. From this model, a protective acetate plate was made to protect the area in the immediate postoperative period, and give more comfort and safety to the patient. This plate was kept for 30 days (Fig 9).

### ORTHODONTIC FOLLOW-UP

At this time, after extraction of the chosen premolar, a fixed space maintainer was installed, preventing tipping of the first permanent molar. The space closure would be performed when orthodontic treatment with fixed appliance begins. This treatment would consist of using a conventional orthodontic appliance with a mini-implant for mesialization of teeth #46, #47 and #48. The patient wore Hyrax maxillary expansion appliance and facemask, and is currently wearing a conventional orthodontic appliance (MBT), in the alignment and leveling phase.

### FOLLOW-UP OF AUTOTRANSPLANTED TOOTH

The proposal for clinical follow-up consists of performing periapical radiographs every 3 months in the first year, a CT scan at 6 months, and serial periapical radiographs annually, for up to 5 years.

**Figure 10:**
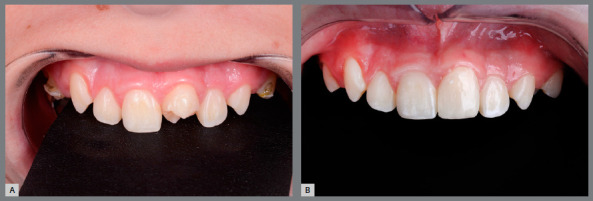
A) Mandibular second premolar positioned at the maxillary central incisor site. B) Immediately after the reshaping of the premolar into a central incisor.

Reshaping of the transplanted tooth was performed one year and three months after the surgical procedure. The cases performed within this protocol were reshaped with a minimum of 12 months after the surgical procedure. This time is longer than that found in most of the literature. We consider that the restoration procedure leads to some degree, even if small, of trauma to the transplanted tooth, and therefore we postponed whenever possible this reshaping (Fig 10).

**Figure 11:**
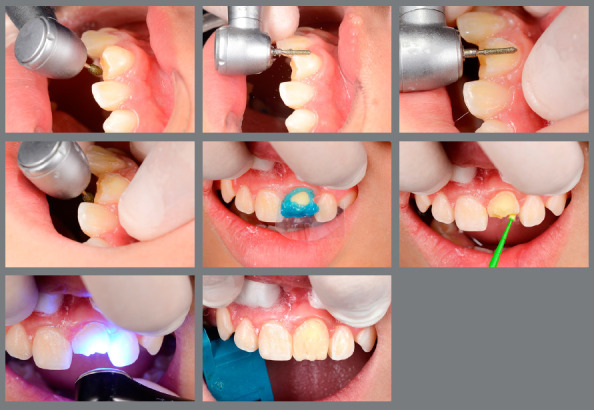
Reshaping and restoration of the transplanted tooth.

The restorative process is performed in a conventional manner, using acid etching, adhesive and light-cured composite resin. An initial wear of the buccal and palatal aspects is performed with a diamond bur, without previous local anesthesia, for better reshaping (Fig 11).

At each post-transplantation consultation, an evaluation of the success and survival of autotransplanted tooth is performed. We followed the control parameters for autotransplanted teeth defined by Shahbazian et al.^17^

The following clinical parameters should be evaluated at each control visit: sensitivity, color, mobility, percussion sensitivity, percussion tone, probing depth and gingival status of the transplanted tooth, in addition to radiographic parameters used to evaluate signs of canal obliteration, overall status of the periradicular area, root length and growth, crown-to-root ratio and root resorption.

**Figure 12:**
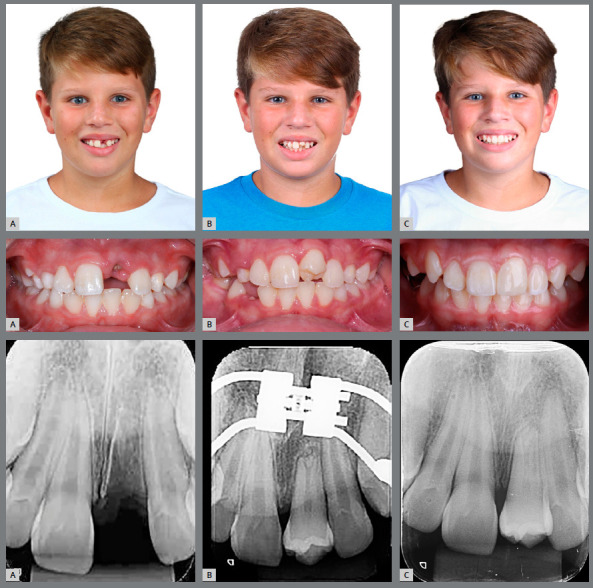
Photographic and radiographic control: A) initial; B) post-surgical; C) post-restoration.

A successful autotransplantation is defined as: the transplanted tooth has normal clinical and radiographic findings, with absence of ankylosis, no progressive resorption or infection; a crown-to-root ratio close to normal; normal mobility and gingival contour; a good level of fixation and normal gingival pocket depth (Fig 12). An autotransplantation is defined as a failure when: at control, the tooth has already been extracted, when it has an unacceptable clinical appearance, with persistent mobility, ankylosis, progressive resorption or infection.

Autotransplanted tooth survival is defined as the transplanted tooth still being present at that control appointment, with or without meeting success criteria.

Table 4 presents these clinical parameters for the present patient at one year postoperative. Radiographic evaluations were also performed at the same time.

**Table 4:** Record of survival and success parameters collected one year (highlighted in bold) after performing the transplantation.

Transplantation clinical control				
Presence of the dental element	YES		No	
Pulp test	NORMAL	Delayed	No response	
Tooth color	NORMAL	Small discoloration	Large discoloration	
Mobility	NORMAL	Abnormal mobility < 1mm	Abnormal mobility > 1mm	Abnormal axial mobility
Percussion test	PHYSIOLOGICAL		Metallic	
Gingival pocket depth	PHYSIOLOGICAL POCKET DEPTH UP TO 3 MM		Pathological	
*Gingival bleeding index*	*Presence of bleeding after 15 seconds of probing*			
Buccal			No	
Distobuccal			No	
Distolingual	YES			
Lingual			No	
Buccolingual			No	
Mesiobuccal			No	

## CONSIDERATIONS

The success rate of the autotransplantation procedure is well defined in the literature. The introduction of virtual planning and prototyping techniques has contributed to increasing this rate and, consequently, to the consolidation of this technique. 

It was possible to observe, by this case report and in the casuistry of the authors, that the use of images obtained by CBCT, in addition to making the surgical explanation and simulation more accessible to the patient, make the diagnosis and preoperative treatment plan more reliable. It is possible to improve the predictability of the procedure, the selection of the donor tooth and create a stereolithographic replica, which allows the dental surgeon to verify surgical feasibility, plan the new position of the donor tooth, maximizing aesthetics and function. Using the replica, a better individual bone adaptation and a reduction of the donor tooth extra-alveolar time are possible. Such improvements assist in preserving the viability of the pulp and periodontal ligament, reducing the risk of necrosis and resorption. Different authors^18^ ^-^ ^22^ reached these same conclusions in their studies. 

The use of clinical parameters of success and survival for the control of autotransplants, as defined by Shahbazian et al.,^17^ as well as periodic radiographic evaluation were applied to this case, with the aim of standardizing these evaluations by creating a standard protocol, thus preventing detection biases, in addition to providing, in the future, the possibility of systematic reviews and meta-analyses based on these and other reports using the same parameters. The proposed clinical follow-up of cases consists of performing periapical radiographs every three months in the first year, a CT scan at six months, and serial periapical radiographs annually for up to five years.

According to Zachrisson et al.,^23^ the following critical factors must be considered in the surgical procedure: the donor tooth extra-alveolar time, and both the skill and experience of the surgeon are very important. Damage to the periodontal ligament should be avoided, because this can lead to ankylosis. The presence of adequate space on the mesial and distal sides of the transplanted tooth, the prevention of occlusal interferences (oscillating contacts) between the transplanted tooth and opposing teeth during the first two months, and physiological mobility during the fixation period are prerequisites for this success. 

The case reported here was performed by an experienced surgeon using an atraumatic technique, and an acetate plate was used postoperatively in order to protect the transplanted tooth against oscillating occlusal contacts in the first month after the procedure. In addition, conventional sutures were preferred for fixation, allowing this physiological mobility during the fixation period.

According to Zachrisson et al.,^23^ one sign of a successful autotransplant is the continuation of root development. As the root of an autotransplanted premolar continues to develop and a normal periodontal ligament is established, these teeth can be moved orthodontically like any other tooth that has erupted and is in occlusion. In general, it is recommended to wait an observation period of three to four months before starting the use of orthodontic forces on these teeth.

A constant criticism of the autotransplantation procedure is the fact that a tooth loss occurs (in this case, the donor tooth) since it will replace the lost tooth, and this loss can lead to malocclusion development. 

With this in mind, this study highlights the key role of the orthodontist in the diagnosis, planning and execution of autotransplantation procedures. The introduction of skeletal anchorage (mini-implants and miniplates) made the mesialization of these molars possible, more predictable and, consequently, likely to be used in routine orthodontic planning. The success of autotransplantation lies in the composition of a multidisciplinary team consisting of a surgeon, an endodontist and an esthetic specialist, coordinated by an orthodontist. 

Orthodontic treatment involving transplanted teeth is part of the success of this treatment proposal, and is reported in some studies^23^ ^,^ ^24^ ^,^ ^25^. A statistically significant prevalence of trauma to maxillary anterior teeth in the mixed dentition was already reported in patients with increased overjet, with or without adequate lip coverage^5^. 

The orthodontist’s role is to make the diagnosis and establish the treatment plan aiming not only to replace a lost tooth, but to obtain a physiological and stable occlusion in the long term. 

## CONCLUSION

From the present case report, it is possible to conclude that autotransplantation is a viable option for the replacement of teeth lost by trauma in the mixed dentition. The technique for this procedure is sensitive, but can lead to a high success rate, especially if virtual planning using prototypes is used. A multidisciplinary approach should be adopted, to achieve the best result for the patient - mainly, the role of the orthodontist to obtain a physiological and stable occlusion in the long term should be emphasized. Randomized clinical trials should be the focus of future studies.

When we take into account the treatment options available for children with anterior tooth loss and the impact of these alternatives on the psychosocial context in which this individual is inserted, we believe that autotransplantation is not only a treatment alternative, but the only viable option for the functional, aesthetic and social reinsertion of these patients.

The results of increasingly frequent transdisciplinary orthodontic treatments of complex cases seem to effectively maximize aesthetic and functional results, by using a combination of procedures conducted by specialists in related areas, such as Surgery, Prosthetics, Implantology, Restorative Dentistry and Periodontics.^26^
